# Converging Evidence for Differential Specialization and Plasticity of Language Systems

**DOI:** 10.1523/JNEUROSCI.0851-20.2020

**Published:** 2020-12-09

**Authors:** Kshipra Gurunandan, Jaione Arnaez-Telleria, Manuel Carreiras, Pedro M. Paz-Alonso

**Affiliations:** ^1^BCBL. Basque Center on Cognition, Brain and Language, 20009 Donostia-San Sebastian, Spain; ^2^Basque Foundation for Science, 48013 Bilbao, Spain; ^3^Department of Basque Language and Communication, University of the Basque Country, 48015 Bilbao, Spain

**Keywords:** bilingualism, comprehension, hemispheric specialization, language learning, laterality, production

## Abstract

Functional specialization and plasticity are fundamental organizing principles of the brain. Since the mid-1800s, certain cognitive functions have been known to be lateralized, but the provenance and flexibility of hemispheric specialization remain open questions. Language is a uniquely human phenomenon that requires a delicate balance between neural specialization and plasticity, and language learning offers the perfect window to study these principles in the human brain. In the current study, we conducted two separate functional MRI experiments with language learners (male and female), one cross-sectional and one longitudinal, involving distinct populations and languages, and examined hemispheric lateralization and learning-dependent plasticity of the following three language systems: reading, speech comprehension, and verbal production. A multipronged analytic approach revealed a highly consistent pattern of results across the two experiments, showing (1) that in both native and non-native languages, while language production was left lateralized, lateralization for language comprehension was highly variable across individuals; and (2) that with increasing non-native language proficiency, reading and speech comprehension displayed substantial changes in hemispheric dominance, with languages tending to lateralize to opposite hemispheres, while production showed negligible change and remained left lateralized. These convergent results shed light on the long-standing debate of neural organization of language by establishing robust principles of lateralization and plasticity of the main language systems. Findings further suggest involvement of the sensorimotor systems in language lateralization and its plasticity.

**SIGNIFICANCE STATEMENT** The human brain exhibits a remarkable ability to support a vast variety of languages that may be acquired at different points in the life span. Language is a complex construct involving linguistic as well as visual, auditory, and motor processes. Using functional MRI, we examined hemispheric specialization and learning-dependent plasticity of three language systems—reading, speech comprehension, and verbal production—in cross-sectional and longitudinal experiments in language learners. A multipronged analytic approach revealed converging evidence for striking differences in hemispheric specialization and plasticity among the language systems. The results have major theoretical and practical implications for our understanding of fundamental principles of neural organization of language, language testing and recovery in patients, and language learning in healthy populations.

## Introduction

Functional specialization in the brain is a well established principle of neural organization, but studies of atypical development suggest dramatic potential for neural plasticity (Payne and Lomber, 2001; Bavelier and Neville, 2002). While the capacity for neural reorganization decreases with age, it does not disappear completely, and adult neural plasticity is essential for learning and maintaining new information or behaviors (Kleim and Jones, 2008). The human propensity for language requires a delicate balance between neural specialization and capacity for reorganization, making language learning the ideal candidate for the examination of specialization and plasticity in the human brain.

Language typically activates a fronto-temporo-parietal network (Skeide and Friederici, 2016; Hagoort, 2019) and has long been thought to be predominantly left lateralized (Broca, 1863; Dax, 1863). However, the right hemisphere appears to be capable of taking over or supporting language function if needed, as seen in cases of language recovery after left hemisphere damage (Papanicolaou et al., 1987; Boatman et al., 1999; Duffau et al., 2002, 2003; Hope et al., 2017) and in language learning (Vingerhoets et al., 2003; Park et al., 2012). It is thus unclear whether the left hemisphere is indeed specialized for language, as is broadly accepted, with the right hemisphere playing at best a supporting role (Vigneau et al., 2011), or whether hemispheric dominance is more variable across individuals, as suggested by the larger than expected prevalence of language deficits following right hemisphere brain surgery (Vilasboas et al., 2017).

Language is a complex construct involving multilevel representations that can be processed visually (reading) or auditorily (listening), or by motor production (speaking/writing), and cumulative evidence points to these functions lateralizing differently. Auditory language has been found to be bilateral in infants (Dehaene-Lambertz et al., 2002; Perani et al., 2011), with no increase in lateralization from childhood to adulthood (Lidzba et al., 2011), increasing left lateralization (Ahmad et al., 2003), or increasing right hemisphere involvement (Booth et al., 2000); and a meta-analysis of auditory comprehension studies suggested that any left lateralization from childhood to adulthood increases more slightly and gradually than previously thought (Enge et al., 2020). On the other hand, there is little evidence to suggest that language production is anything but left lateralized (Gaillard et al., 2003; Szaflarski et al., 2006; Lidzba et al., 2011).

Language learning is known to change the pattern of neural activation for language. Studies comparing bilinguals and monolinguals consistently find differences in activation between them, with bilinguals typically exhibiting greater right hemispheric involvement in comprehension tasks (Kovelman et al., 2008; Horowitz-Kraus et al., 2015). However, it is uncertain whether this increased right hemispheric involvement merely modulates the magnitude of left lateralization or whether it is significant enough to constitute a change in hemispheric dominance. Further, are differences in lateralization between monolinguals and bilinguals because of developmental differences or is hemispheric dominance in fact plastic even into adulthood? Few neuroimaging studies have looked into ecologically valid adult language learning, but findings indicate that language learning in adults involves structural changes in cortical thickness and connectivity that could indeed support shifts in lateralization (Mårtensson et al., 2012; Schlegel et al., 2012; Xiang et al., 2015), suggesting that lateralization, at least for comprehension, may be susceptible to learning-dependent changes.

We conducted two fMRI experiments, one cross-sectional and one longitudinal, with immersed late language learners, and examined lateralization of reading, speech comprehension, and verbal production in their native (L1) and non-native (Ln) languages, and how this changed with increasing Ln proficiency. To test both the replicability and generalizability of findings, the two experiments were contrasted on several factors such as the early language experience of the participants (monolingual vs bilingual) and the language currently being learned, and the L1–Ln pairs in the two experiments had contrasting degrees of overlap in language families, phonology, and orthography. We hypothesized that (1) lateralization of comprehension would be more variable across individuals but production would be left lateralized; and (2) with increasing language proficiency, comprehension may display changes in hemispheric dominance, while production would remain left lateralized. We further expected that L1–Ln associations would change with increasing Ln proficiency, and that the pattern of changes would differ across the language systems.

## Materials and Methods

### 

#### Participants

##### Experiment I: basic versus advanced level language learners (cross-sectional)

The final experiment sample consisted of 29 right-handed native Spanish adults (mean age = 43.7 ± 9.7 years; 15 female) studying Basque in the same language school at either the basic (A2 level, *n* = 14) or advanced level (C1 level, *n* = 15). The proficiency levels correspond to those specified by the Common European Framework of Reference for Languages (CEFR). Participants were from the Basque Country, Spain; they grew up primarily exposed to Spanish at home and in school, with little early Basque exposure, and had limited knowledge of English or other languages (no difference between groups, *p* = 0.83). The two groups of learners were matched on age, gender, IQ, and Spanish proficiency (Table 1). Data from five other participants were discarded because of excessive head motion during MRI scanning, and these were not counted in the final sample.

**Table 1.** Participant demographics and linguistic scores

	Experiment I			Experiment II		
	Basic proficiency group	Advanced proficiency group	Statistical tests	Before training	After training	Statistical tests
Age	42.9 (10.1)	44.5 (10.5)	*t*_(26.9)_ = 0.44, *p* = 0.66, two-sample *t* test	17.2 (0.6)		
Gender	7 female, 7 male	8 female, 7 male	χ^2^ (1) = 0, *p* = 1 χ^2^ test for independence	16 female, 3 male		
L1 Proficiency	99.35 (1.88)	99.64 (0.77)	*t*_(13.9)_ = 0.52, *p* = 0.61, two-sample *t* test	99.11 (1.49)	99.26 (1.15)	*t*_(23)_ = 0.90, *p* = 0.56, paired *t* test
Ln Proficiency	52.6 (14.66)	87.96 (10.58)	*t*_(19.4)_ = −7.02, *p* = 0.0000009, two-sample *t* test, Cohen's *d* = 2.82	58.00 (11.73)	62.89 (12.82)	*t*_(23)_ = 2.98, *p* = 0.006, paired *t* test, Cohen's *d* = 0.42

Values correspond to the mean with SD in parentheses.

##### Experiment II: intermediate language learners (longitudinal)

The final experimental group consisted of 19 right-handed native Spanish adolescents (mean age = 17.2 ± 0.6 years; 16 female) taking part in a 3 month English immersion-style after school program for B1 level students. Participants were from the Basque Country, Spain; they were native speakers of Spanish and acquired Basque in school (age of acquisition = 2.6 ± 2.06 years). The medium of instruction in school was Spanish/Basque; English was learned as a foreign language, with little exposure outside of classes. The students had intermediate English proficiency (Table 1). Data from five other participants were discarded because of excessive head motion during MRI scanning, and these were not counted in the final sample.

##### Experiments I and II

In both experiments, language proficiency was assessed using picture-naming tasks—an adaptation of the Boston Naming Test (Kaplan et al., 1983) controlled for cognates across Spanish, Basque, and English. Participant groups in Experiment I differed significantly in their Basque proficiency, and participants in Experiment II exhibited a significant increase in English proficiency after language training (Table 1). All participants had normal or corrected-to-normal vision, and no history of neurologic or psychiatric disorders. In compliance with the ethical regulations established by the Basque Center on Cognition, Brain and Language (BCBL) Ethics Committee and the guidelines of the Helsinki Declaration, all participants gave written informed consent before taking part in the experiment and received monetary compensation for their participation.

#### fMRI task

Inside the MRI scanner, participants performed the following two tasks: a comprehension and a production task. The order of tasks was counterbalanced across participants.

##### Language comprehension task

The participants performed semantic animacy judgment (living/nonliving) with single-word text and speech stimuli in each of their languages. Participants were instructed to fixate on a white cross in the middle of a black screen, and on the presentation of stimuli, to indicate their responses as quickly and as accurately as possible via button presses (counterbalanced across participants) using their dominant (right) hand. Stimuli were high-frequency, concrete, imageable nouns (e.g., house, dog, table) with an even split between living and nonliving items. Visual stimuli were presented in white letters on a black screen and were five to eight letters long. Auditory stimuli were presented through headphones and lasted an average of 565 ms (SD = 86 ms). Each run had 48 stimuli with intermixed reading and listening trials. The fMRI design was event-related with six/four runs (Experiment I: 2 languages × 3 runs; Experiment II: 2 languages × 2 runs). To avoid language switching, the languages were separated and their order was counterbalanced across participants.

##### Language production task

The participants performed a paced form of the semantic verbal fluency task in each language. Participants were instructed to fixate on a white cross in the middle of a black screen and respond overtly to semantic category words (e.g., fruits, animals, clothes) presented on the screen. Each word was displayed eight times, each requiring a novel response, or, failing this, an overt response saying “pass” in the relevant language. Fluency was scored as the percentage of valid answers of eight possible responses for each category. Repetitions, inflections of the same word and erroneous responses were removed, and responses were scored only for correctness and not accent or pronunciation. In the control condition, participants repeated the word presented on the screen. The task had a block design with two runs per language, each run containing eight semantic categories. To avoid language switching, the languages were separated and their order was counterbalanced across participants.

#### MRI data acquisition

Whole-brain MRI data were acquired using a 3 T Siemens Magnetom Trio whole-body MRI scanner and a 32-channel head coil at the BCBL. Padded headphones were used to dampen background scanner noise and enable clear transmission of the auditory stimuli. Participants viewed the print stimuli on a screen via a mirror mounted on the head coil. To limit head movement, the head coil was padded with foam and participants were asked to remain as still as possible. Structural T1-weighted images were acquired with an MPRAGE sequence [repetition time (TR) = 2530 ms, echo time (TE) = 2.97 ms, inversion time = 1100 ms, flip angle (FA) = 7°, field of view (FOV) = 256 × 256 mm, 176 slices and voxel size = 1 mm^3^].

##### Language comprehension task

Functional MRI was acquired in the course of six/four separate runs using a gradient echo echoplanar pulse sequence with the following parameters: TR = 2000 ms, TE = 30 ms, 32 axial slices with a 3.4 × 3.4 × 4 mm voxel resolution, 0% interslice gap, FA = 80°, FOV = 220 mm, 64 × 64 matrix. A total of 186 volumes were collected in each of the functional runs. Before each scan, four volumes were discarded to allow for T1 equilibration effects. To improve estimation of the resting baseline in functional analyses, functional runs contained three silent fixation periods of 20 s each. Within each functional run, the order of the trials (reading and listening conditions) and the intertrial intervals of variable duration corresponding to the baseline MR frames (30% of total collected functional volumes) were determined by an algorithm designed to maximize the efficiency of the recovery of the blood oxygenation level-dependent response (optseq2; Dale, 1999).

##### Language production task

Functional MRI was acquired in the course of four separate runs using a gradient echo echoplanar pulse sequence with the following parameters: TR = 3000 ms, TE = 25 ms, 43 axial slices with a 3.0 × 3.0 × 3.0 mm voxel resolution, 10% interslice gap, FA = 90°, FOV = 192 mm, 64 × 64 matrix. Two hundred forty volumes were collected for each of the functional runs. Before each scan, four volumes were discarded to allow for T1-equilibration effects.

#### MRI data analysis

##### Preprocessing

Standard SPM8 (Penny et al., 2011) preprocessing routines and analysis methods were used. Images were first corrected for differences in the timing of slice acquisition and then realigned to the first volume using rigid-body registration. Each subject's functional volumes were spatially smoothed with a 4 mm full-width at half-maximum (FWHM) Gaussian kernel. Next, motion parameters obtained from realignment were used to inform a volume repair procedure (ArtRepair; Mazaika et al., 2009) that identified bad volumes on the basis of scan-to-scan movement (>1 mm) and signal fluctuations in global intensity (>1.3%), and that corrected bad volumes via interpolation between the nearest nonrepaired scans. Data from subjects requiring >20% of volumes to be repaired were discarded. The number of corrected volumes was similar between groups (Experiment I: comprehension task, *p* = 0.34; production task, *p* = 0.63) and scans (Experiment II: comprehension task, *p* = 0.75; production task, *p* = 0.46). After volume repair, functional volumes were coregistered to the T1 images using 12-parameter affine transformation and spatially normalized to the MNI space by applying nonlinear transforms estimated by deforming the MNI template to each individual's structural volume. During normalization, the volumes were sampled to 3 mm cubic voxels. The resulting volumes were then spatially smoothed with a 7 mm FWHM Gaussian kernel. Finally, time series were temporally filtered to eliminate contamination from slow frequency drift (high-pass filter with a cutoff period of 128 s).

##### Subject-level analyses

Statistical analyses were performed on individual subject data using the general linear model (GLM). fMRI time series data were modeled by a series of impulses convolved with a canonical hemodynamic response function (HRF). Six motion parameters for translation (*x*, *y*, *z*) and rotation (yaw, pitch, roll) were included as covariates of noninterest in the GLM. In the event-related design comprehension task, each trial was modeled as an event that was time locked to the onset of the presentation of each stimulus, and error responses were modeled separately. In the block design production task, each trial was modeled as an epoch of 31 s each, time locked to the beginning of the presentation of each block. The remaining functions were used as covariates in the GLM, along with a basic set of cosine functions that high-pass filtered the data, and a covariate for session effects. The least-squares parameter estimates of the height of the best fitting canonical HRF for each experimental condition were used in pairwise contrasts.

##### Laterality analyses

For every subject, lateralization of activation in the classical language network regions was calculated for each task × language. Laterality is typically quantified as a normalized ratio of left and right hemisphere contributions, ranging between +1 (fully left-lateralized activation) and −1 (fully right-lateralized activation). Each subject's whole-brain *t*-maps were masked with anatomic language regions from the AAL atlas (Tzourio-Mazoyer et al., 2002)—six bilateral regions from classical language models (Friederici, 2012; Hagoort, 2013): inferior frontal gyrus (IFG) pars orbitalis, IFG pars triangularis, IFG pars opercularis, superior temporal gyrus, middle temporal gyrus, and inferior parietal lobule. Since laterality indices are highly threshold dependent, in line with the latest recommendations (Bradshaw et al., 2017a), a threshold-independent bootstrapping method was used to calculate the laterality index using the LI-toolbox (Wilke and Lidzba, 2007), in which 10,000 indices were iteratively calculated at different thresholds, yielding a robust mean laterality index. Three analyses were conducted to examine the proficiency-dependent plasticity of (1) L1–Ln correlation, (2) hemispheric dominance, and (3) modality clustering. L1–Ln correlations were calculated for each group × task, and Cohen's *q* was used to quantify the difference in L1–Ln correlation between basic/advanced proficiency and before/after training in each modality. To examine hemispheric dominance, a lateralized dissociation index was calculated such that:

Lateralized Dissociation Index=LIL1-LILn * hem

hem=1 ifopposite lateralization-1 if same lateralization

that is, the absolute difference between laterality indices for each language and a factor, hem, to code whether the two languages were lateralized to the same or opposite hemispheres. Positive values indicated that languages were lateralized to opposite hemispheres, while negative values indicated that the languages were lateralized to the same hemisphere. Cohen's *d* was used to measure the magnitude of proficiency-dependent change in each modality: difference between medians in cross-sectional Experiment I, and difference in repeated measures in longitudinal Experiment II. To examine the modality-wise clustering of the joint L1–Ln distribution, 85% data ellipses were plotted for each modality, and the joint distribution difference (JDD) between any two modalities was calculated as:

JDD=distance between centroidsmaximum distance * angle between major axesmaximum angle,

that is, standardized difference between the bivariate L1–Ln group means and the difference between joint spread of the data. This index lies between 0 and 1, with higher values indicating a greater difference between modalities. The maximum Euclidean distance between centroids was considered to be 1 for laterality data, and the maximum angle between the axes is 90°. Proficiency-group differences were measured in terms of the percentage difference in the difference index.

## Results

### In-scanner behavioral performance

#### Experiment I: basic versus advanced level language learners (cross-sectional)

**Figure 1.**
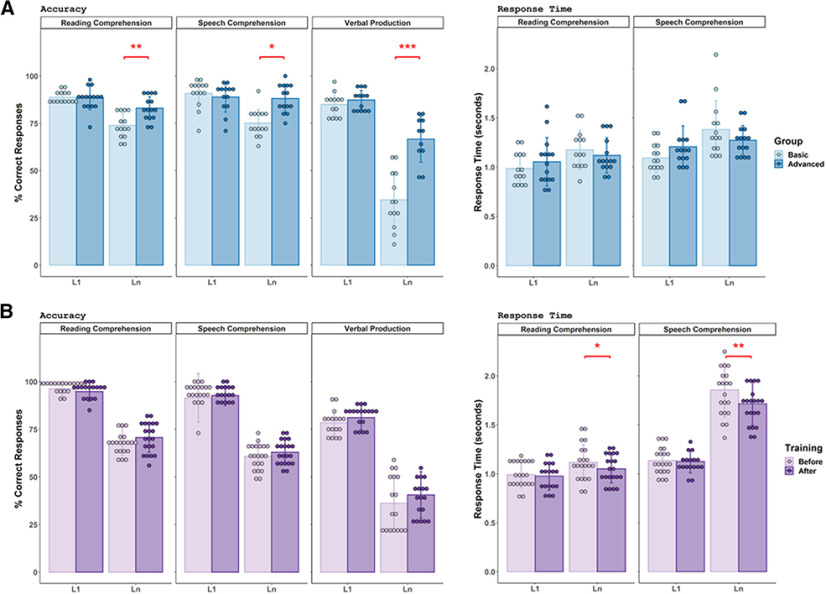
***A***, ***B***, Behavioral measures accuracy and response time for in-scanner semantic tasks plotted as a function of Group, Language, and Modality in Experiment I (***A***), and as a function of Training, Language, and Modality in Experiment II (***B***). Error bars represent the SD, and asterisks represent statistically significant differences (***p < 0.001, **p < 0.01, *p < 0.05).

A series of mixed-model ANOVAs was conducted on the behavioral measures of the fMRI tasks: comprehension task accuracy, production task fluency, and comprehension task reaction times (Fig. 1*A*). The comprehension task accuracy ANOVA with between-subjects factor Group (basic, advanced) and within-subject factors Language (L1, Ln) and Modality (reading, speech) showed a significant Group × Language interaction (*F*_(1,26)_ = 16.18, *p* = 0.0004). The production task fluency ANOVA with between-subjects factor Group (basic, advanced) and within-subjects factor Language (L1, Ln) also showed a significant Group × Language interaction (*F*_(1,23)_ = 31.36, *p* = 0.00,001). *Post hoc* simple-effect analyses (two-sample *t* tests) of these Group × Language interactions showed that the advanced proficiency group had significantly higher Ln task accuracy than the basic proficiency group in both comprehension (*t*_(18.08)_ = 3.20, *p* = 0.002, one-sided) and production (*t*_(22.28)_ = 5.502, *p* = 0.000008, one-sided), but there was no significant difference between groups in L1 task accuracy (comprehension: *t*_(25.48)_ = −0.93, *p* = 0.360, two-sided, production: *t*_(21.683)_ = 1.03, *p* = 0.31, two sided). Finally, the ANOVA for comprehension task reaction times showed a main effect of Language, and both groups were significantly slower in their Ln than their L1 (*F*_(1,26)_ = 40.41, *p* = 0.000001).

#### Experiment II: intermediate language learners (longitudinal)

A series of repeated-measures ANOVAs was conducted on the following behavioral measures of the fMRI tasks: comprehension task accuracy, production task fluency, and comprehension task reaction times (Fig. 1*B*). The comprehension task ANOVAs with three within-subject factors, Training (before, after), Language (L1, Ln), and Modality (reading, speech), showed the main effects of Language (L1 > Ln, *F*_(1,17)_ = 338.64, *p* = 0.000000000001) and Modality (reading > speech, *F*_(1,17)_ = 30.05, *p* = 0.00,004) on task accuracy. The production task fluency ANOVA with two within-subject factors Training (before, after), and Language (L1, Ln) showed a main effect of Language (L1 > Ln, *F*_(1,15)_ = 146.01, *p* = 0.000000004). The comprehension task reaction times ANOVA revealed a significant Training × Language interaction (*F*_(1,17)_ = 5.48, *p* = 0.031). *Post hoc* simple-effect analyses (paired *t* tests) showed that reaction times decreased significantly after training in Ln (*t*_(17)_ = 2.83, *p* = 0.006, one-sided), but not in L1 (*t*_(17)_ = 0.21, *p* = 0.836, two sided).

### Language lateralization

#### Lateralization in comprehension and production

**Figure 2.**
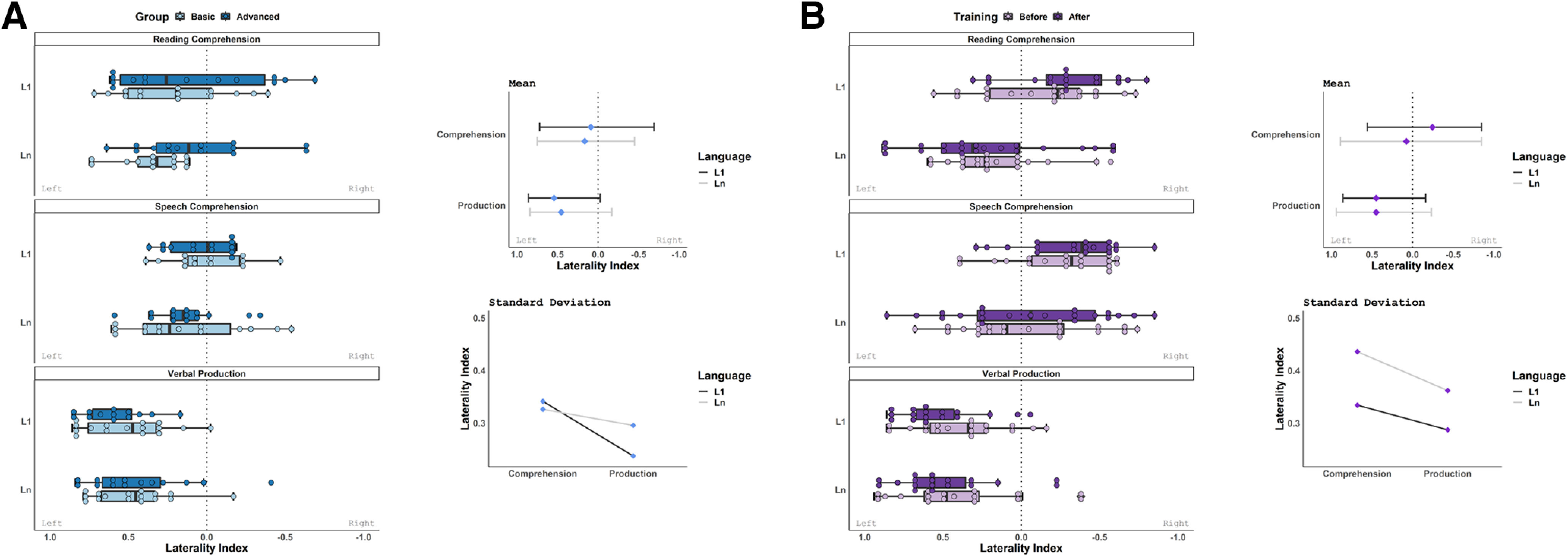
***A***, ***B***, Laterality indices plotted as a function of Group, Language, and Modality in Experiment I (***A***), and Training, Language, and Modality in Experiment II (***B***). Laterality indices were obtained from individual whole-brain activation in the neuroanatomical language network, and the respective line graphs display the mean and SD of laterality indices across participants in each Modality and Language.

Laterality indices were calculated for the language network regions in each task and language using a threshold-free method, with values between +1 (left lateralization) and −1 (right lateralization). In both experiments, Wilcoxon signed-rank tests of paired samples revealed significant differences between each of the modalities. Comprehension and production displayed robust differences in lateralization, with significant differences between both reading and verbal production (Experiment I: W = 421, *p* = 0.000000000006; Experiment II: W = 1099, *p* = 0.0000000004) as well as between speech comprehension and verbal production (Experiment I: W = 824, *p* = 0.000002; Experiment II: W = 729.5, *p* = 0.000000000000006). Reading and speech comprehension also differed significantly (Experiment I: W = 1998.5, *p* = 0.021; Experiment II: W = 3840, *p* = 0.012). In reading and speech comprehension, lateralization was highly variable and indices spanned the full range of possible values between the two languages, while verbal production was clearly left lateralized. At the group level, comprehension appeared bilateral and production was left lateralized. This result was consistent across the cross-sectional and longitudinal experiments (Fig. 2).

#### Learning-dependent changes in lateralization

**Figure 3.**
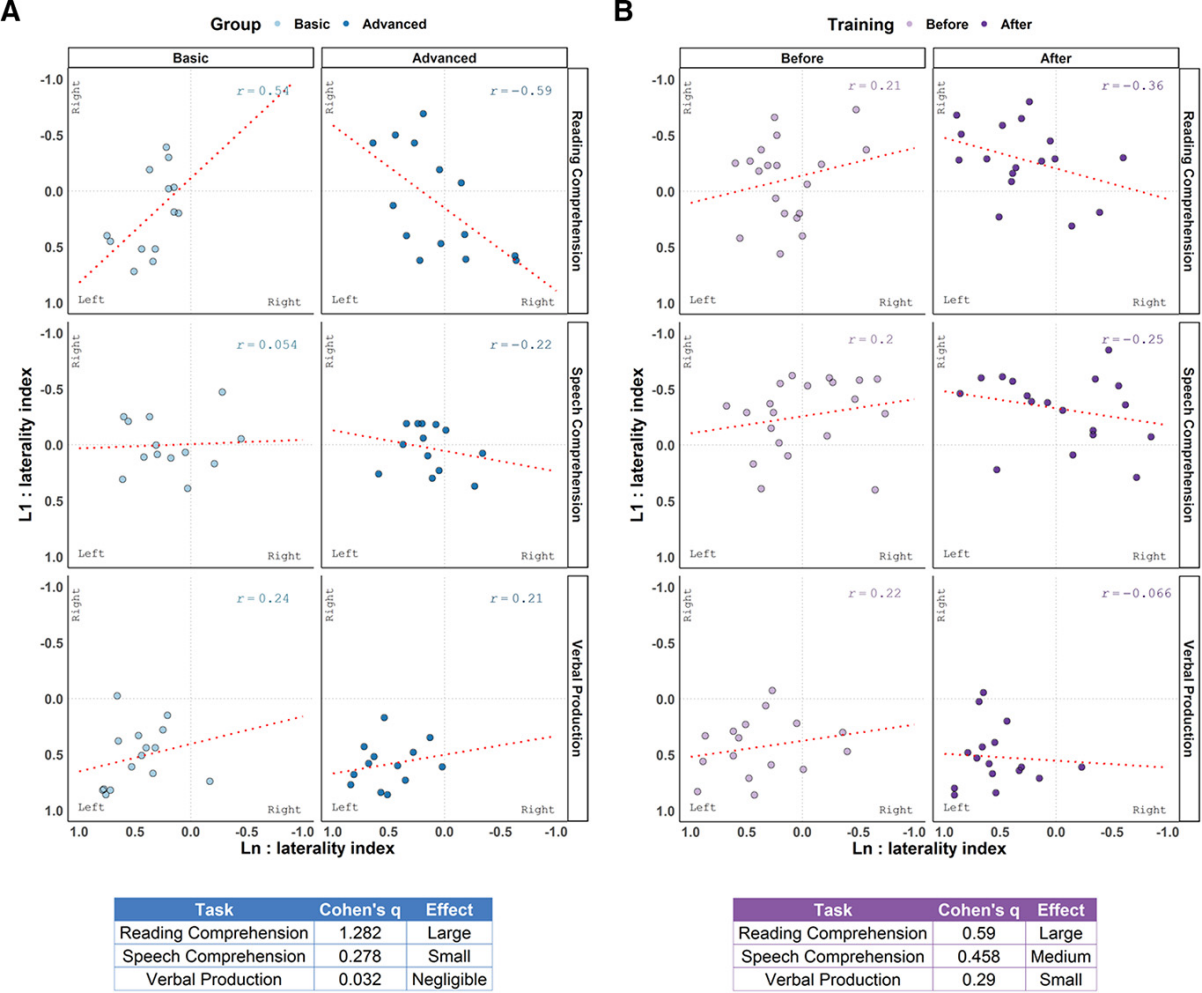
***A***, ***B***, Linear associations between L1 and Ln lateralization indices (Pearson's *r*) as a function of Group and Modality in Experiment I (***A***) and of Training and Modality in Experiment II (***B***). Cohen's *q* quantified the learning-dependent changes in L1–Ln correlation in each modality.

To examine patterns of learning-dependent changes in lateralization while accounting for the high interindividual variability across tasks and languages, L1 lateralization was used as a baseline for each subject's Ln lateralization, and the linear association between L1 and Ln was assessed using Pearson's *r*. In lower-proficiency learners, L1 and Ln lateralized similarly, regardless of left/right lateralization. However, with increasing proficiency, this pattern reversed for comprehension, and L1 and Ln lateralized to opposite hemispheres. This learning-dependent change was not observed in verbal production (Fig. 3). Cohen's *q* was used to quantify the proficiency-dependent change in L1–Ln correlation for each task, confirming that, across both studies, learning-dependent change in lateralization was large in reading comprehension, medium in speech comprehension, and small in verbal production.

**Figure 4.**
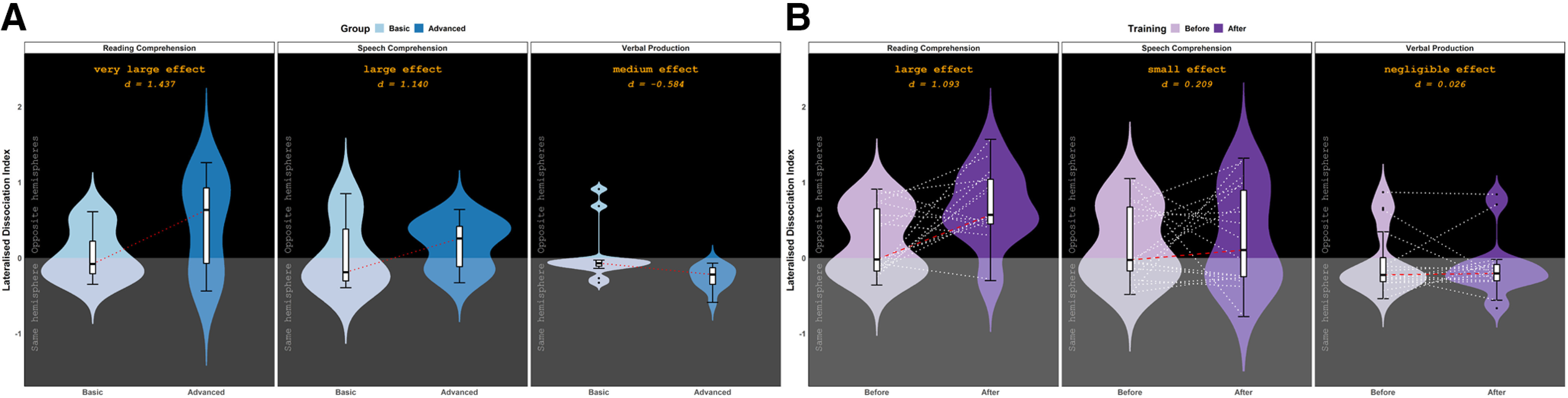
***A***, ***B***, Lateralized dissociation indices (LDIs) as a function of Group and Modality in Experiment I (***A***), and Training and Modality in Experiment II (***B***). Positive values indicate that L1 and Ln lateralized to opposite hemispheres, and negative values indicate that L1 and Ln lateralized to the same hemisphere. Cohen's *d* quantified the learning-dependent changes in LDI in each modality. Error bars represent SD.

To examine whether increasing proficiency involved changes in hemispheric dominance for each modality, lateralized dissociation indices were calculated for each subject such that absolute values indicated the magnitude of L1–Ln difference, and direction (i.e., positive or negative) indicated whether the languages were lateralized to the same or opposite hemispheres (positive = opposite hemispheres, negative = same hemisphere). There was a significant proficiency-related increase in absolute dissociation between L1 and Ln lateralization across modalities (Experiment I: Mann–Whitney *U* tests: across modalities: W = 584.5, *p* = 0.013; reading comprehension: W = 41, *p* = 0.007; speech comprehension: W = 88, *p* = 0.579; verbal production: W = 53.5, *p* = 0.022; Experiment II: Wilcoxon signed-rank tests: across modalities: V = 374.5, *p* = 0.023; reading comprehension: V = 15, *p* = 0.004; speech comprehension: V = 53, *p* = 0.142; verbal production: V = 78, *p* = 0.330), and Cohen's *d* was used to quantify learning-dependent change in hemispheric dominance for each modality. In both experiments, the same pattern of changes emerged: large in reading comprehension, medium in speech comprehension, and small in verbal production (Fig. 4).

**Figure 5.**
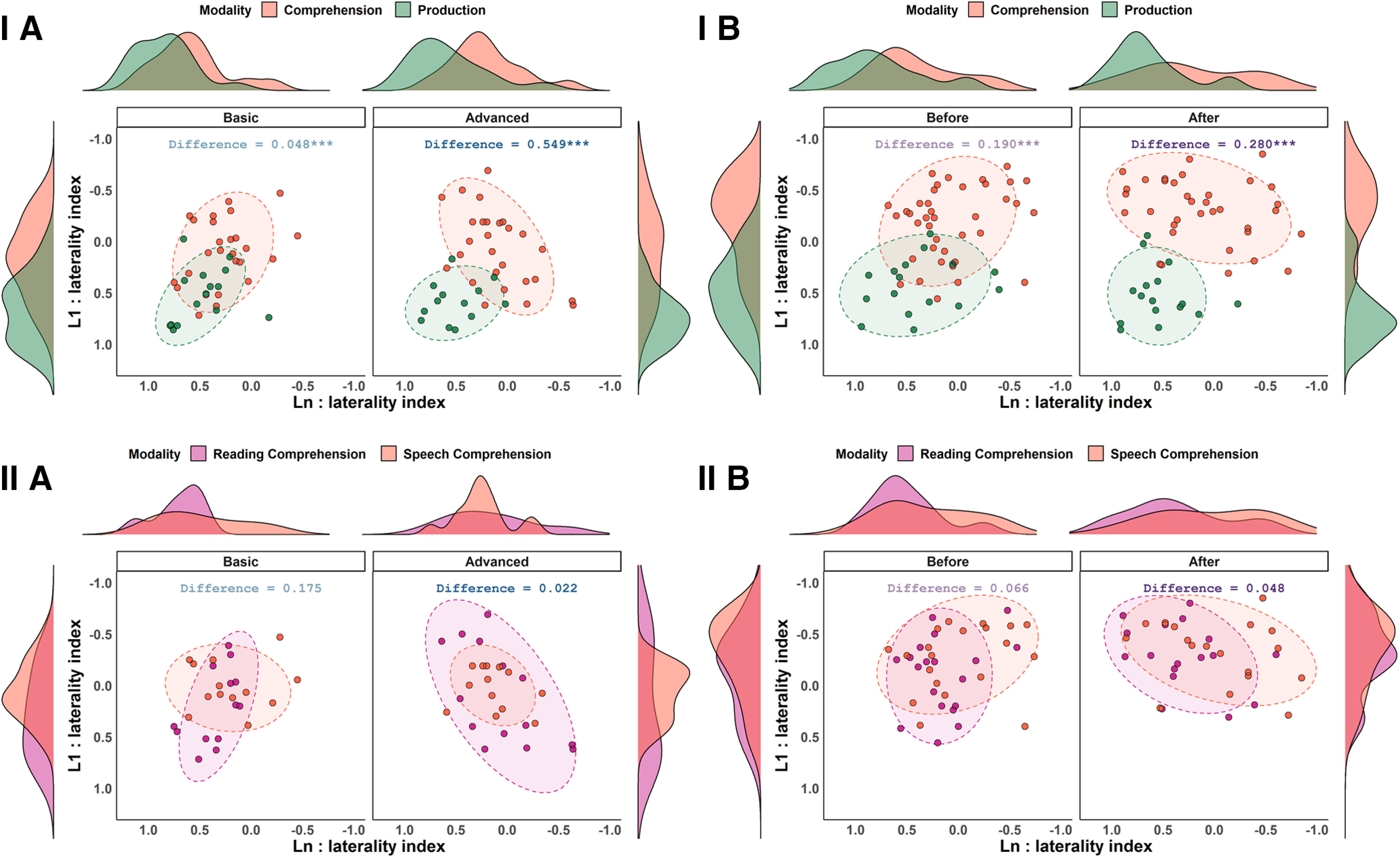
***IA–IIB***, Modality-wise clustering (comprehension vs production, ***I***; reading vs speech comprehension, ***II***) in joint distributions of L1–Ln lateralization indices plotted as a function of Group in Experiment I (***A***) and as a function of Training in Experiment II (***B***). A joint distribution difference index with values between 0 and 1 quantified overlap in each group, with higher values indicating larger separation between modalities. Asterisks represent statistically significant differences (*p* < 0.001).

Finally, modality-wise clustering of joint L1–Ln lateralization was plotted using 85% data ellipses to examine overlap between modalities. One-way multivariate ANOVAs (MANOVAs) and a joint distribution difference index were used to test and quantify the separation between the following: (1) comprehension (both reading and speech) and production (Fig. 5*I*); and (2) reading and speech comprehension (Fig. 5*II*). The effects of proficiency were tested using nonparametric two-sample/paired tests of difference/change in cluster separation between modalities (Euclidean distance) and quantified with the percentage change in the JDD. The one-way MANOVA modeled the joint L1–Ln distribution differences between modalities, and the index quantified this difference by taking into account the difference in both bivariate mean and spread of data, with values between 0 (overlapping distributions) and 1 (no similarities). MANOVAs revealed significant differences between comprehension and production (Experiment I: basic proficiency group: *F*_(1.8,65.2)_ = 11.73, *p* = 0.0005; advanced proficiency group: *F*_(1.9,63.8)_ = 22.96, *p* = 0.00000002; Experiment II: before training: *F*_(1.7,73.2)_ = 21.67, *p* = 0.0000002, after training: *F*_(1.7,70.7)_ = 38.94, *p* = 0.0000000000004), and with increasing proficiency, comprehension, and production dissociated further (Experiment I: the advanced proficiency group displayed 1042.35% greater comprehension–production dissociation than the basic proficiency group, Mann–Whitney *U* test of group difference in cluster separation: W = 67 398, *p* = 0.000000000003; Experiment II: participants displayed 47.38% increase in comprehension–production dissociation after training, Wilcoxon signed-rank test of post-training change in cluster separation: V = 101769, *p* = 0.0000000000000002). There were no significant differences in L1–Ln joint distribution between reading and speech comprehension (Experiment I: basic proficiency group: *F*_(1.9,45.5)_ = 1.84, *p* = 0.18; advanced proficiency group: *F*_(1.7,41.4)_ = 0.32, *p* = 0.71; Experiment II: before training: *F*_(1.9,71.2)_ = 1.98, *p* = 0.15; after training: *F*_(1.8,60.5)_ = 2.09, *p* = 0.13), and reading and speech comprehension converged further with increasing proficiency (Experiment I: the advanced group displayed 87.27% greater comprehension–production overlap than the basic group, Mann–Whitney *U* test of group difference in cluster separation: W = 18073, *p* = 0.177; Experiment II: participants displayed 27.13% increase in comprehension–production overlap after training, Wilcoxon signed-rank test of post-training change in cluster separation: V = 39 306, *p* = 0.0005).

## Discussion

In the present work, we examined hemispheric specialization and learning-dependent plasticity of the language network concurrently in three language systems: reading, speech comprehension, and verbal production. We conducted cross-sectional and longitudinal fMRI experiments in separate populations of immersed language learners. Both experiment samples had the same L1 (Spanish), but were contrasted in other factors: (1) early language experience: monolingual versus sequential bilingual; (2) language being learned: Basque versus English; (3) phonological similarity with native language: high overlap versus low overlap; and (4) orthographic depth: transparent versus opaque. Across these contrasting experimental designs and participant groups, we found a highly consistent pattern of results in both experiments: (1) across native and non-native languages, lateralization for language comprehension was variable but language production was strongly left lateralized; and (2) with increasing non-native language proficiency, reading and speech comprehension displayed significant changes in hemispheric dominance (reading > speech), while verbal production remained left lateralized. The converging results from separate experiments provide unique insight into the long-standing debate on hemispheric specialization of language and the effects of language experience (Gainotti, 1993; Price, 1998, 2012; Jung-Beeman, 2005; Hickok and Poeppel, 2007; Friederici, 2012; Hervé et al., 2013; Tzourio-Mazoyer et al., 2017).

The first result showing variably lateralized (bilateral at the group-level) comprehension and left lateralized verbal production across different languages suggested that comprehension is flexible while verbal production is hardwired to be left lateralized. Previously, conflicting evidence from studies in monolinguals had led to a range of different conclusions and models of comprehension: from left lateralized to partly bilateral, bilateral, or right-lateralized function (Booth et al., 2000; Gaillard et al., 2003; Jung-Beeman, 2005; Hickok and Poeppel, 2007; Lidzba et al., 2011). Few studies have compared different modalities in the same participants, and, though lateralization was seen to be highly modality-dependent in the current study, it did not appear to depend on the exact task used, since lateralization for the single-word overt tasks in the current study was consistent with results from far more complex discourse-level covert tasks in previous studies (Dehaene et al., 1997; Lidzba et al., 2011; Bhattasali et al., 2019). There were also subtle differences between the two experiments, with similar Ln lateralization but differing central tendencies for L1 laterality. This pattern is consistent with the literature on the influence of early language experience: meta-analyses have found that early bilinguals (L2 acquired before age 6 years) typically show bilateral hemispheric involvement, while monolinguals and late bilinguals show greater left hemisphere dominance (Hull and Vaid, 2006, 2007; Bloch et al., 2009; Liu and Cao, 2016). Thus, the convergent results in the present work indicate that interindividual variability in lateralization for language comprehension is not an artifact of task or methodology, but that instead, language comprehension is differently lateralized across individuals. Lesion studies in children have found dissociative effects of lesion side on comprehension and production: while lesions in the left hemisphere were associated with more severe delays in production compared with comprehension, comprehension delays were more common—but not universal—in children with right hemisphere damage (Marchman et al., 1991; Bates, 1993; Thal et al., 1991). In line with these findings, developmental neuroimaging studies all found left-lateralized language production, but reached conflicting conclusions on comprehension, leading to a suggestion of differing maturational mechanisms for comprehension and production (Hervé et al., 2013). Clinical studies have recommended that both comprehension and production tasks be used in determining language lateralization for clinical purposes (Wilke et al., 2010; Lidzba et al., 2011; Vilasboas et al., 2017; Woodhead et al., 2018). Modality-dependent lateralization (i.e., variably lateralized comprehension vs left-lateralized production) could explain the long-standing conflicts among previous studies that used tasks tapping into different modalities and shed new light on the question of functional specialization for language.

Our analytic approach to examining learning-dependent changes in language lateralization built on the observed interindividual variability and used within-subject measures calculated with each subject's L1 as a baseline for their Ln. We used three measures—L1–Ln correlation, L1–Ln distance, and modality clustering—and quantified the change within each language system. These revealed that (1) L1 and Ln were similarly lateralized in lower-proficiency language learners and tended to dissociate with increasing Ln proficiency; (2) the change was largest in reading, smaller in speech comprehension, and smallest in verbal production; and (3) with increasing proficiency, comprehension, and production dissociated, while reading and speech comprehension converged.

Convergence and dissociation of neural activation for different languages and language systems has been of considerable research interest. Neuroimaging studies of language have by and large come to the conclusion that all languages do indeed recruit the same language regions, and that language experience modulates the amount of overlap, leading to the “convergence hypothesis” (Perani and Abutalebi, 2005; Gurunandan et al., 2019). The current study built on this finding, and characterized lateralization patterns for L1 and Ln within the common language network, finding that increasing Ln proficiency led to increasing dissociation in lateralization between the two languages. There has been much debate on whether language control in bilinguals is language specific or domain general, with mixed evidence (Hernández et al., 2013), and it is possible that, apart from any changes in the involvement of language control regions, the greater hemispheric separation of languages in more proficient nonmonolinguals also contributes to their improved language control. Future studies looking concurrently at dissociation within the language network and recruitment of language control regions are needed to test this idea. Comprehension and production also dissociated with increasing Ln proficiency. In lower-proficiency learners, there was lower separation between modalities, possibly indicating variable strategies of Ln access and variable activation profiles (Dehaene et al., 1997), but, as individuals attained higher proficiency, their activation profiles stabilized and became more uniform. Turning to the question of convergence between language systems, print–speech convergence has been considered a universal signature of native language proficiency (Shankweiler et al., 2008; Rueckl et al., 2015; Preston et al., 2016), but it is less well studied in multilinguals. In the current study, we found increasing convergence of joint L1–Ln lateralization for reading and speech comprehension with increasing language learning, suggesting that reading–speech convergence is also sensitive to increasing Ln proficiency.

The pattern of plasticity differences between the language systems (i.e., plasticity for reading > speech comprehension > verbal production) was strikingly similar to their perceived difficulty in real-world language learning in adults. Two observations support the idea that the differential plasticity of language systems contribute to differential learning. First, learners in the longitudinal study had switched languages from same to opposite hemispheres in reading within a relatively short time frame, while fewer did so for speech comprehension, and none for production. Further, individuals who had L1 and Ln lateralized in opposite hemispheres maintained this dissociation post-training, and individuals who had L1 and Ln in the same hemisphere tended to dissociate post-training to varying degrees depending on the modality. This suggested that opposite hemispheric dominance of languages could be advantageous for language learning, and, further, that shifts in hemispheric dominance are limited by the plasticity of the sensory/motor cortices corresponding to each language system. Neuropsychological evidence from stroke recovery patterns in adults who showed greater (but not complete) recovery in comprehension than in production (Lomas and Kertesz, 1978), as well as different reorganization patterns for comprehension and production (Musso et al., 1999; Heiss and Thiel, 2006) further supports our conclusion. Though the visual, auditory, and motor cortices are all bilateral, each of them exhibits hemispheric advantages for processing specific features (Benke and Kertesz, 1989; Deruelle and Fagot, 1997; Flinker et al., 2019; Albouy et al., 2020), and previous studies with monolinguals have found differences in visual lateralization of different writing scripts (Tzeng et al., 1979; Kuo et al., 2001), asymmetrical sensitivity of the auditory cortices (Friederici and Alter, 2004; Boemio et al., 2005), and left lateralization of auditory and articulatory motor areas (Morillon et al., 2010), pointing to differential potential for post-critical period plasticity of these sensory/motor regions that matches the converging pattern of language system plasticity found in the current study. Second, the adolescent learners in the second experiment displayed substantial neural changes after just 3 months of training, while the adult learners in the first experiment displayed similar neural differences for a much larger proficiency difference between groups. This finding is compatible with age-related decrease in neural plasticity and sheds further light on the source of the difficulty of late language learning. However, despite the convergence of the neural results in Experiments I and II, the modest behavioral effect in Experiment II limited any further interpretation of the neural changes in relation to behavioral outcomes at the individual level in naturalistic language learning. In sum, taken together with previous evidence, the converging findings in the present work point to the sensorimotor cortices playing a large role in both the lateralization of language as well as the asymmetric decrease in plasticity of the language network.

Methodological studies and reviews of language lateralization have often warned against the overinterpretation of results from a single task, small regions of interest, or nonrobust analytical methods (Bradshaw et al., 2017a,b; Bain et al., 2019). These were avoided in the current study, and interpretations were based on the robust patterns of results verified by corroborating analyses that were replicated in contrasting experiments. However, the current study used classical single-word tasks, and while the lateralization results were consistent with the findings from far more complex comprehension tasks (Dehaene et al., 1997; Lidzba et al., 2011; Bhattasali et al., 2019), future studies are needed to establish whether the results presented in the current study would be as or possibly even more pronounced in sentence/discourse processing (Hagoort, 2019). Further, a priori power analysis was not conducted, nor was a replication sample examined. The two experiments involved ecologically valid language learning, and the lateralization results were sensitive to participants' real-world language-learning progress (i.e., CEFR level) rather than their performance or improvement on the in-scanner semantic tasks involving high-frequency stimuli. In fact, while performance on the tasks was relatively uniform across participants, lateralization exhibited much larger variation in both languages, supporting the idea of multifactorial modulation of hemispheric specialization (Tzourio-Mazoyer et al., 2017), since participants were carefully selected to control for language backgrounds as much as possible, but actual experimental control on early or previous language exposure was not possible. The replication of findings in language learners at different levels of proficiency suggested that the learning-dependent neural changes were not temporary, but further studies are necessary to disentangle the effects of learning versus proficiency, and test lateralization of languages in early balanced bilinguals. Finally, the two experiments featured distinct L1–Ln language pairs that were contrasted on factors such as overlap in language families, and phonological and orthographic properties, but did not involve more sensory differences such as visual differences between writing systems or auditory perception and motor production of tones, as in, say, English–Chinese. Following from our idea that the sensorimotor cortices are the limiting factor in language learning and its associated neural changes, it is possible that late acquisition of a language that requires greater sensorimotor learning would entail smaller proficiency-dependent neural changes in lateralization and the size of the changes would decrease more sharply with increasing age than in the current study.

In conclusion, our study design with cross-sectional and longitudinal experiments in contrasting samples of real-world language learners, testing of different language systems, and a multipronged analytical approach revealed robust and converging patterns of modality-dependent lateralization and plasticity of the language network. Our findings suggest that language lateralization for reading and speech comprehension is plastic well into adulthood, while production shows strong left hemisphere specialization. Plasticity for reading was greater than for speech comprehension, which was in turn greater than for verbal production. Together with previous evidence in the literature, we propose that hemispheric specialization for language may arise from the sensorimotor cortices, and that the differential plasticity of language systems is tied to the plasticity of the associated sensorimotor systems.
